# Event-Triggered Ephemeral Group Communication and Coordination over Sound for Smart Consumer Devices

**DOI:** 10.3390/s19081883

**Published:** 2019-04-20

**Authors:** Doohwan Song, Ikjun Yeom, Honguk Woo

**Affiliations:** 1 Samsung Electronics, Suwon 16677, Korea; duhwan.song@samsung.com; 2 Sungkyunkwan University, Suwon 16419, Korea; ikjun@skku.edu

**Keywords:** voice-based interface, hotword, coordinated react, ephemeral group, data communication over sound, smart consumer device

## Abstract

Voice-based interfaces have become one of the most popular device capabilities, recently being regarded as one flagship user experience of smart consumer devices. However, the lack of common coordination mechanisms might often degrade the user experience, especially when interacting with multiple voice-enabled devices located closely. For example, a hotword or wake-up utterance such as “hi Bixby” or “ok Google” frequently triggers redundant responses by several nearby smartphones. Motivated by the problem of uncoordinated react of voice-enabled devices especially in a multiple device environment, in this paper, we discuss the notion of an ephemeral group of consumer devices in which the member devices and the transient lifetime are implicitly determined by an external event (e.g., hotword detection) without any provisioned group structure, and specifically we concentrate on the time-constrained leader election process in such an ephemeral group. To do so: (i) We first present the sound-based multiple device communication framework, namely *tailtag*, that leverages the isomorphic capability of consumer devices for the tasks of processing hotword events and transmitting data over sound, and thus renders both the tasks confined to the same room area and enables the spontaneous leader election process in a unstructured group upon a hotword event. (ii) To improve the success rate of the leader election with a given time constraint, we then develop the adaptive messaging scheme especially tailored for sound-based data communication that inherently has low data rate. Our adaptive scheme utilizes an application-specific score that is individually calculated by a member device for each event detection, and employs score-based scheduling by which messages of a high score are scheduled first and so unnecessary message transmission can be suppressed during the election process. (iii) Through experiments, we also demonstrate that, when a hotword is detected by multiple smartphones in a room, the framework with the adaptive messaging scheme enables them to successfully achieve a coordinated response under the given latency bound, yielding an insignificant non-consensus probability, no more than 2%.

## 1. Introduction

Theses days, voice-based interfaces and assistants (e.g., Google Assistant, Apple Siri, Amazon Alexa, and Samsung Bixby) have obtained attention from the consumer device markets. Besides high-end smartphones equipped with voice-based interfaces and several auxiliary sensors, a set of new voice-enable devices of different form factors, e.g., Home speaker [1] and Familyhub refrigerator [2], have been introduced, and even resource-constrained wearables and smartwatches have become more capable of natural interactions. It is definitely anticipated that non-traditional smart devices such as connected cars and home appliances (e.g., robot cleaners) will render their user interfaces more intelligent and context-aware with ever-evolving machine learning based cognitive applications as well as device components including microphones and speakers. The global market of voice assistants is expected to grow significantly, up to USD 7.8 billion by 2023 [3], and the competition in the market gets fiercer; e.g., at the Bixby developer event in 2018, it was announced that Samsung plans to install the voice assistant Bixby on all its consumer electronics products, i.e., billions of devices, by 2020 [4].

**Figure 1:**
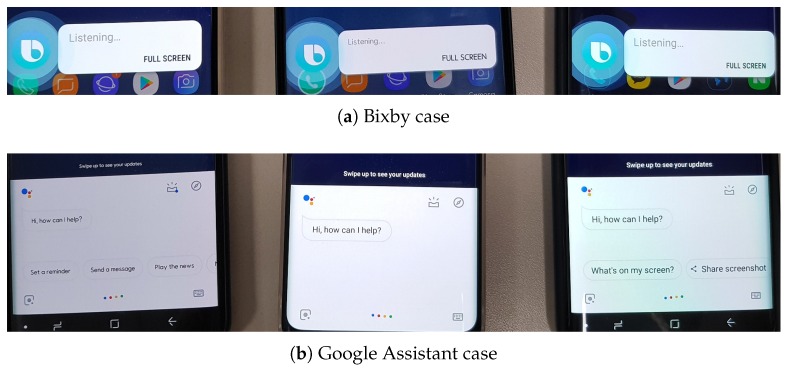
Uncoordinated react of voice-based interaction.

As the trend of natural interactions is likely to expand across various device types and service domains, it might necessitate the cautious configuration and deployment in order not to incur awkward situations and unnecessary communication. Consider the common case in which two or more smartphones having the same voice assistant are closely located. Having a hotword (wake-up), e.g., “ok Google” or “hi Bixby”, a user might experience that several simultaneous responses, often seemingly arbitrary, from different smartphones are activated as the react signals (see Figure 1).

Few workaround schemes for achieving a controlled and consensus decision about a responder among multiple devices are introduced. The short phrases for hotwords can be customized [5] or the priority among a set of devices being logged-in under the same account can be stored [6], which commonly relies on managed backend infrastructures and user configurations, or  only considers single-user scenarios. Similar to the aforementioned case of desiring a single coordinated response of multiple consumer devices upon a user hotword, there are several situations in which a set of devices in close proximity often need to instantly build a group and then temporally collaborate for taking a timely step toward the given group goal, e.g., sharing the credentials upon a new member in an ad-hoc network and tracking objects by surveillance sensors and cameras [7,8,9]. We consider a group of devices to be *ephemeral* when an external event *implicitly* sets up the group members only for a short period while the event is being handled by the group. Thus, an ephemeral group is temporal and *no provision* at the infrastructure level for the group management is assumed.

In this paper, we focus on such an ephemeral group being triggered by a hotword event in a room, and investigate its coordination mechanism tailored for timely reacting to the event. To this end, we first present the sound-based multiple device communication framework, *tailtag* that renders data transmission naturally confined to the same room area with hotword detection, and  thus supports short group communication with no group structure. Notice that sound-based communication is effective for applications with room area context [10]. Accordingly, having a hotword event in a room, the *tailtag* framework enables each device in the event-triggered ephemeral group to efficiently broadcast its message in the group. Furthermore, we address the time-constrained leader election problem over sound by exploring adaptive messaging strategies that can improve the quality of the *tailtag* framework in terms of the success rate of timely leader election.

Note that the aforementioned problem of uncoordinated react of multiple voice-enabled consumer devices can be investigated within the scope of the traditional leader election problem in a distributed system, which has been studied over several decades. Formal algorithms are proposed to solve the problem in [11,12,13], and they are applied to practical areas such as mobile ad-hoc networks [14] and wireless sensor networks [15].

The leader election problem for our ephemeral group setting, however, is more practical and requires a specific solution under the following two conditions: (i) to maintain the same room area context between event detection and group communication, devices utilize sound for data transmission; and (ii) to meet the user interaction requirements of voice-based interfaces, the time bound of each election process is strictly restricted. Under these two conditions, we develop an adaptive messaging scheme that can be seen as a variant of bully algorithms [13,16], yet adapted for sound-based data communication and time-constrained leader election. Specifically, having an application-specific score that is immediately available upon event detection, each device adjusts the backoff delay value depending on its score in a way that messages of a high score are delivered first. This behavior of individual devices reduces collisions on shared sound channels especially for important messages and thus is likely to increase the chance to resolve the leader within a given time bound. The enhancements of adaptive messaging are discussed and presented in the form of several adaptive delay functions in Section 3.3.

The proposed framework combined with the adaptive messaging scheme enables us to incorporate a lightweight coordination mechanism into voice-based interfaces (e.g., Bixby on Samsung smartphones), thereby enhancing voice-based user experiences especially in a multiple device environment. Moreover, the isomorphic structure of the framework and existing voice-based interfaces simplifies the framework integration at device-side. The contributions of this paper are as follows.

- Having the motivating real-world problem, that is, the uncoordinated reaction of today’s voice-enabled consumer devices (in Section 2.1), we present the framework solution *tailtag*, which employs the sound-based data communication tailored to the problem (in Section 3).
- We introduce the adaptive messaging scheme, considering the inherent restriction of sound for data transmission. This enables expediting the message transmission of high significance and suppressing the unnecessary message transmission, hence providing a reliable solution to our specific time-constrained leader election problem (in Section 3.3).
- We conducted simulation experiments under various environmental conditions, demonstrating that the framework adoption is able to meet the requirement of the real application scenarios, such as 98% accuracy for the Bixby hotword case, where the accuracy was measured based on the rate that, within the given latency bound, a single (non-redundant) response among multiple Bixby-enabled smartphones is appropriately made (in Section 4).

## 2. Background

In this section, we explain the motivation of our work toward the sound-based communication and coordination for an ephemeral group of consumer devices and describe the technical foundation of short messaging over sound wave.

### 2.1. Motivation

Suppose there are three smartphones with the voice-based service, e.g., D1 and D2 of user U1 and D3 of user U2 in near proximity. Further, suppose that user U1 tries activating the service with its hotword. It is expected that either D1 or D2 responds, but often they both respond redundantly and even worse D3 does too. We call these undesirable situations *uncoordinated react*.

Figure 1 depicts the example situations of uncoordinated react by hotword utterances of Bixby and Google Assistant, showing that three Android smartphones all are simultaneously responding to the hotword “hi Bixby” or “ok Google”, respectively. It is observable that the situations happen mainly due to two conditions: (i) the limited capability of individual devices in context understanding; and (ii) no mechanism commonly adopted for device collaboration.

**Figure 2:**
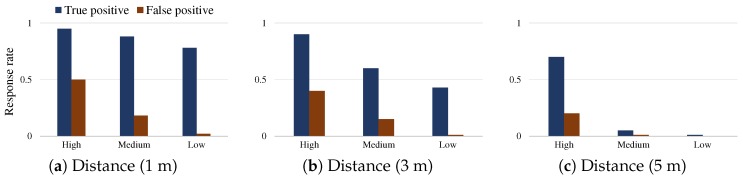
Tradeoff of Sensitivity Configuration for Hotword Response: (**a**) the response rates (on *y*-axis) depending on the sensitivity options (on *x*-axis) when a hotword is uttered at 1 m distance; and (**b**,**c**) the cases of 3 m and 5 m distance, respectively. The test was performed 100 times per each configuration (combination) of three sensitivity options, five testers (adults aged over 25, with about 70 dB utterance volume), and three different distances in a room (5 m${}^{2}$) with no significant ambient noise, less than 40 dB.

Figure 2 demonstrates an example of the limited capability of an individual device in recognizing the user context and determining whether it should respond to a user hotword. We tested the Bixby voice assistant app on Samsung Galaxy S8 Android smartphones, which has the advanced *sensitivity* configuration such that the low sensitivity intends to cautiously react only to the registered user (i.e., the smartphone owner who registered and trained her voice), thus frequently rendering no response at all, while the high sensitivity intends to react anyway, thus frequently incurs unwanted redundancy. This recently released sensitivity supports the user-configurable settings, including low, medium, and high options, and aims at mitigating the issue of limited context understanding such as inherently imperfect recognition of the registered user voice. However, our tests with the options show the tradeoff. In the 1 m distance test between a tester saying the Bixby hotword and the two smartphones (one is registered by the tester and the other is not), when having the medium sensitivity, about 87% hotword requests of the registered tester were successfully responded (denoted by True positive rate, $\frac{the\;number\;of\;tester\;device^{\prime }s\;responses}{the\;number\;of\;tester^{\prime }s\;hotwords}$), but many requests (up to 19%) were arbitrarily responded (denoted by False positive rate, $\frac{the\;number\;of\;the\;other\;device^{\prime }s\;responses}{the\;number\;of\;tester^{\prime }s\;hotwords}$); similarly, when having the low (high) sensitivity setting, both percents decreased (increased). Notice that the the response rate for each sensitivity option might vary depending on the experiment conditions and thus our analysis should be limited to only the tradeoff pattern commonly observed with respect to the sensitivity options. Notice that the sensitivity of the current Bixby version needs to be manually set by a user, and, furthermore, it rarely considers a multiple device environment, thus has no support for configuring a set of devices of a same user or multiple users.

The undesirable behaviors of individual devices aforementioned become even worse in multiple user, multiple device environments. Thus, it is worthwhile considering a common coordination mechanism for enabling a group of individual devices to shortly communicate and collaborate, hence, e.g., letting them achieve a single response appropriately through some consensus process upon an event toward the group. For building such a common mechanism of device communication and collaboration, it is natural to exploit either cloud-based service or device-to-device direct connectivity approaches. In the cloud-based service approach, each device with long-distance connectivity acts as a client connecting to a server and so client devices in a group can communicate and collaborate by the direction of the server. In the direct device-to-device approach, local connectivity such as BLE or WiFi is exploited for discovery, pairing, and sharing among nearby devices. In the following, we explain that both conventional approaches might not be a good solution for the the problem of uncoordinated react unless correctly configured for a specific scenario.

**Figure 3:**
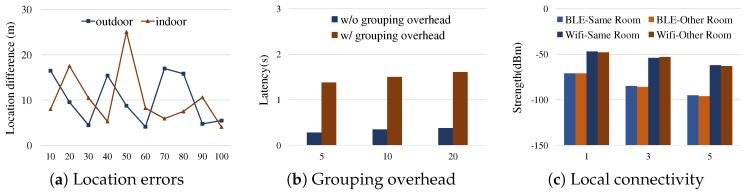
Challenges of conventional cloud-based and local connectivity approaches for the uncoordinated react problem: (**a**) the difference of location estimates (on *y*-axis) of the two smartphones of a same walking person over time, based on the 10 measurements at every 10 s during 100 s (on *x*-axis); (**b**) the latency of group information sharing (on *y*-axis) with respect to the group size (on *x*-axis), where the grouping overhead denotes the case where no provisioned group connections are managed, thus requiring the time for establishing the group connection upon a request; and (**c**) the measured strength averages (on *y*-axis) of BLE and WiFi signals with respect to the distances between the signal source and the receiver (on *x*-axis) where the measurement was performed 100 times for each distance and connectivity. All tests were performed with Samsung Galaxy S8 and S6 smartphones.

In cloud-based services, a group of devices needs to be provisioned based on their proximity or user registration. The GPS position accuracy of mobile devices has been investigated and measured as, e.g., 5–8.5 m range errors outdoors [17], and up to 100 m range errors indoors [17]. Figure 3a illustrates the location estimation accuracy of smartphones both indoors and outdoors. In this experiment, we set up a pair of Samsung Galaxy devices to update the location estimates using Android 8.0 location APIs (android.location.LocationManager) every 10 s to the server, and then observe the high variances. This implies that grouping a set of devices based on the location APIs is not appropriate particularly when the devices are in close proximity.

While there are enhanced technologies on indoor localization, e.g., WiFi RSS fingerprinting [18,19,20] that relies on the mapping of data collected before operation, or trilateration [21] that exploits distance measurements from the reference locations by multiple access points [18], those require a certain type of system provisioning at the infrastructure level. Figure 3b illustrates the overhead of device group management and communication through cloud severs. For the experiment, we set up a group of 5–20 clients connecting a provisioned backend server through either WiFi or LTE, and let them immediately share the information upon an external event, a pair of client id and random integer *k*, via the server so that they could render a consensus about the top rank $\arg\max_{id}k$. The latency was increased more than three times unless a group of clients could immediately utilize the established, authenticated connections to the server, and use the provisioned group management.

**Figure 4:**
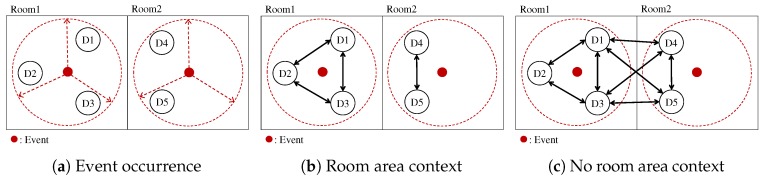
Communication in Room Area Context: each circle denotes the confinement of the ephemeral group members in the room area context, but others in the next room might have signals due to the penetrative nature of local connectivity. (**b**,**c**) The cases of having the room area context and not, respectively, for the two events occurred simultaneously, as shown in (**a**).

Most local connectivity technologies have penetrative nature. Thus, the local connectivity only solutions are not often useful for the room area context [10]. Figure 3c shows the received signal strength measured by smartphones using BLE and WiFi, indicating that those signals are rarely useful for differentiating the devices in the same room and the devices in the next room. In Figure 4, suppose we expect only D1, D2 and D3 in the same room become the members of a group for the event in the Room 1. As shown in Figure 4c, however, D4 and D5 in the next room might be within the range where the local connectivity is reached, and then there might be no significant difference among the devices in terms of the connectivity signals. Such room area context problems can be addressed by using the sound-based communication since sound wave signals hardly pass through walls and thus only devices in the same space can communicate using the sound wave [10].

The aforementioned limitation of conventional cloud-based and device-to-device approaches necessitates an alternative method to make an instant group and enable the short group communication. In the next section, we explain the generalized form of the problem of uncoordinated react and our approach based on sound-based data communication.

### 2.2. Sound-Based Data Communication

We consider the use cases of today’s smart consumer devices in which an external event occurs in a multiple device environment and a set of devices individually detecting the event need to form a tentative group for collaboratively taking an action. As explained above, such a group is regarded to be ephemeral and it has neither provisioned controllers nor predefined group structures. In the context of uncoordinated react, we view that a hotword is such an external event that triggers an ephemeral group of consumer devices, and a timely, non-duplicated, and appropriate response is such an action of the group. Our proposed approach for supporting an ephemeral group, the *tailtag* framework, employs sound-based data communication. It should be noted that voice-based interfaces and our approach are isomorphic in that processing hotword events and using the *tailtag* framework utilize the same microphone and speaker capability of devices, and both rely on the room area context.

In the following, we show a set of profiling experiments on sound-based data communication with modern consumer devices, Samsung Galaxy S8 (or S6) smartphones that support digital recording up to 32 bits/384 kHz (or 24 bits/192 kHz) and uses a high AOP (Acoustic Overload Point) microphone. Specifically, we implemented the MFSK (Multiple Frequency Shift Keying) modulation using AudioTrack [22] with the setting of 44.1 kHz sampling frequency as the same setting as most audio interfaces and 4410 samples per block. This yielded 0.1 s for the transmission time of one block (denoted by $D_{p}$). We utilized the inaudible frequency range between 18 kHz and 21 kHz, considering the environment of consumer devices directly interacting with users. Furthermore, we established 100 usable frequencies (denoted by $F_{all}$) for the given bandwidth of 3 kHz in that the fast Fourier transform was used to extract the frequency in 30 Hz units when receiving signals.

**Figure 5:**
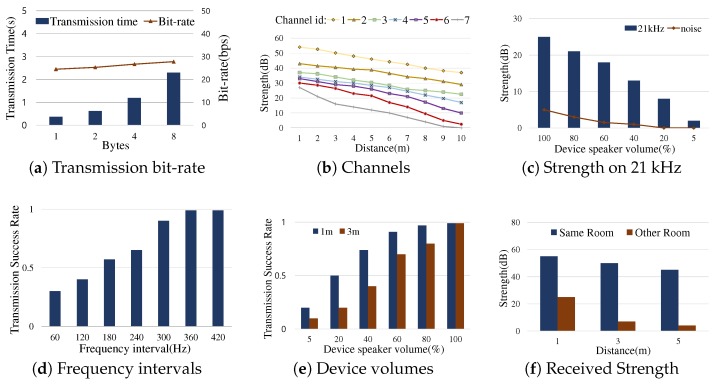
Data transmission experiments using microphones and speakers of consumer devices: these show the test results when MFSK modulation is used for small data transmission through the inaudible frequency range. Each measurement was performed 100 times and denoted by their average value.

Figure 5a depicts the performance characteristic of data transmission over sound in our default profiling configuration where the distance of the sender and the receiver devices was 1 m in a room and the sender device utilized the maximum volume over inaudible sound. While the transmission time varied greatly depending on the byte size (the difference of 1 byte and 4 bytes is more than three times), there was insignificant difference in bit-rate. Having the same setting of the previous experiment, Figure 5b shows the received signal strength over different channels. In this experiment, we had seven channels in a way that the channel id was incremented on the inaudible frequency from 18 kHz to 21 kHz, As shown, the closer to 18 kHz was the channel, the stronger was the received signal. Similarly, Figure 5c depicts the received signal strength and the noise strength on the 21 kHz band (channel id = 7) where the strength was weakest. The result by the different volumes of the sender device allowed us to configure the threshold value, i.e., 5 dB, for appropriately filtering noises.

Figure 5d demonstrates the effect of possible signal interference that can be incurred by the narrow intervals between adjacent frequency bands used. The experiment showed that the 360 Hz interval can ensure the high success rate of transmitting data. Figure 5e shows the effect of device volumes. As expected, it was found that the higher was the device volume, the higher was the success rate of transmitting data. The experiment illustrated that the distance was also relevant because it influenced the strength measured by a receiver. Figure 5f depicts the benefit of sound-based communication by which devices can be properly differentiated in the room area context, different from the other local connectivity technologies shown in Figure 3c.

The experiments thus far motivated us to design the framework optimized particularly for small data transmission with time constraints in a multiple device environment. The framework follows several design choices toward the optimization: (i) We define the short message format for the framework that contains two fields (1-bit start tag, 4-bit data payload). As shown in Figure 5a, the short message structure favors the low latency. (ii) We use MFSK, a simple and robust modulation method [23,24,25,26] to efficiently support small-sized messages. (iii) We exploit the multiple channels over the inaudible frequency range from 18 kHz to 21 kHz. The threshold value for filtering noises is set at 5 dB (Figure 5c). The guard band for mitigating the inter-channel signal interference of FDM is set at 360 Hz (Figure 5d), and thus 12 frequencies of the channel-to-channel guard interval (denoted by $F_{g}$) are obtained.

## 3. Framework Design

In this section, we describe the framework architecture and the framework parameters.

### 3.1. Overall System

**Figure 6:**
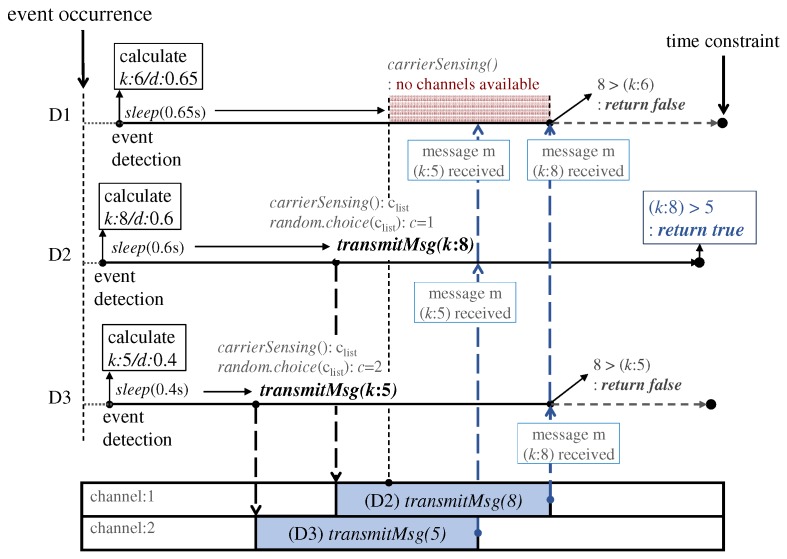
System flow example: As shown in the left figure of Figure 4b, upon an external event occurrence in a room, devices D1, D2 and D3 all detect it simultaneously and independently, and thus the dotted circle denotes the area in which the event can be reached and confines the ephemeral group members. This example assumes two channels. Then, each member device individually follows the steps of the supported framework, as explained in Section 3.1.

Figure 6 depicts the system flow of the *tailtag* framework at each device. A member device in the ephemeral group encapsulates a message of significance score with respect to the event detected, and then broadcasts the message data over sound in the group. As shown below, through the framework, we implemented a variant of bully algorithms [13,16] designed to meet the requirement of our time-constrained leader election scenario of an event-triggered ephemeral group. Specifically, the framework enables reaching a group consensus about the member device responding to the event, i.e., the device of the highest score within a given time bound. In the example, D2 sends its message with the score *k* = 8 on the channel *c* = 1 after some random delay *d* = 0.6 s, and, similarly, D3 sends its message with the score *k* = 5 on the channel *c* = 2. However, D1 performs carrier sensing and cancels its message. Given the time duration (time constraint in the figure), each device can figure out the highest score *k* = 8 of all the messages received yet, and thus is able independently to decide whether to respond to the event; D2 becomes the responder. We use the term *round* to denote the bounded duration for handling an event between the time when the event is detected and the time when the responder is determined. The significance score is encoded in the data field of a message and is used for comparison. The goal of ephemeral group communication is to timely reach a consensus and thus we facilitate the decision process by using the significance score. Specifically, a high-scored message successfully delivered in a group can suppress all the messages with lower scores (e.g., D1’s message in Figure 6). Hence, the framework strategically favors high-scored messages when scheduling messages over a set of shared channels.

Algorithm 1 implements the system flow of each device. When detecting an event, each device first initiates the round of the event by activating the timer of TC, the time constraint associated with the round. The device obtains the message data, its score *k* with respect to the event *e*, and the wait delay *d* from the framework functions $f_{s}\left(e\right)$ and $f_{d}\left(k\right)$, respectively. After waiting for *d* time, the device checks by the carrier sensing routine to retrieve the available channels $c_{list}$. The device then transmits the message on the channel *c*, which is randomly selected among the available ones. Note that e.ts and curtime() denote the event detection time and the current system time, respectively, and they are used to see if the device can send a message successfully within the time constraint.

In parallel, the device receives messages *m* from others and keeps the maximum of *m*’s data (m.k) to get the highest score in the group. If any message higher than its own message is received during the round, the device immediately cancels the timer and terminates the round. Otherwise, upon the expiration of the timer, the device compares the messages received including its own message to determine whether it should become a responder. Note that for the responder decision, the channel on which the message is received is used when having tie scores, and the candidate(k) function is used when neither receiving any message nor transmitting. The default value of candidate(k) is based on the *k*-weighted random, and the statistical evaluation is explained in Section 3.3. In this system flow, we assume the non-overlapping event occurrences within the time duration of each round. The assumption might be a restriction to the framework generality, yet it is consistent with the behavior of the ephemeral group of voice-enabled devices in that, e.g., a set of multiple, simultaneous hotword utterances to a device do not generate the same set of corresponding responses, but normally one or none.

**Algorithm 1** handleEvent.**OUTPUT:** whether or not respond **On event *e*:**1:setupTimer(TC), $k_{max}\leftarrow 0$, $c_{max}\leftarrow 0$, $flag_{send}\leftarrow False$2:$k\leftarrow f_{s}\left(e\right)$, $d\leftarrow f_{d}\left(k\right)$ // calculate score and delay time3:sleep(d)4:**while do**5:    **if** $(c_{list}\leftarrow carrierSensing\left(\right))>0$ **then**6:        $c\leftarrow random.choice(c_{list})$7:        transmitMsg(k) on channel *c*8:        $flag_{send}\leftarrow True$9:        break10:    **end if**11:**end while** **On message *m* from channel $c_{m}$:**1:**if**isTimerAlive(TC)**then**2:    **if** m.k>k **then**3:        cancelTimer(TC) // early termination4:        returnFalse // do not respond5:    **else if** $m.k>k_{max}$ **then**6:        $k_{max}\leftarrow m.k$ // update the highest score7:        $c_{max}\leftarrow c_{m}$8:    **else if** $m.k=k_{max}\wedge c_{m}>c_{max}$ **then**9:        $c_{max}\leftarrow c_{m}$ // keep the channel for the same highest scores10:    **end if**11:**end if** **On timer expiration TC:**1:**if**$flag_{send}=True\wedge k=k_{max}\wedge c\geq c_{max}$**then**2:    returnTrue // respond3:**else if**$flag_{send}=True\wedge k>k_{max}$**then**4:    returnTrue // respond5:**else if**$flag_{send}=False\wedge k_{max}=0$**then**6:    returncandidate(k)7:**else**8:    returnFalse // do not respond9:**end if**

**Figure 7:**
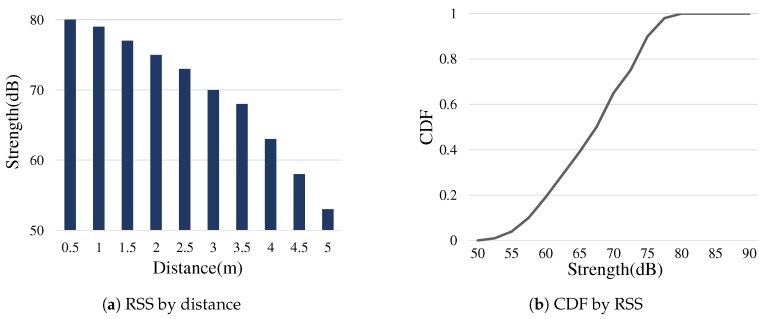
Voice Event Strength: (**a**) the average RSS of smartphones detecting user hotwords with respect to the distance from the user, 0.5 to 5 m; and (**b**) its cumulative distribution. Each measurement was performed 100 times for 10 different distances and two testers.

In the following, we describe the functions of the algorithm. Table 1 illustrates the framework parameters used in our explanation. The score captures the relevance of a device about dealing with the given event. Having the cases of hotword events, we implement a score function with respect to the event distance approximation based on RSS (Received Signal Strength) as

(1) $$f_{s}\left(e\right)=\left\lfloor \frac{\int_{x=0}^{RSS(e)}CDF\left(x\right)}{\int_{x=0}^{\infty }CDF\left(x\right)}\right\rfloor \cdot S$$

where RSS(e) is $20log_{10}\left(amplitude\;of\;e\right)$ and CDF() is the distribution estimated by our experiments on RSS measurements of hotword utterances. Figure 7 shows the the average RSS of normal hotword utterances measured by a smartphone having the same specification for the experiment in Figure 2, with respect to various distances between the user and the smartphone in a room area. Note that the accurate distance measurement using sound signals is beyond the scope of this paper, and the score function above is an example implementation. We rather consider the extensibility of the score function depending application requirements. For instance, the priority setting can be an additional input to the function such that the home speaker might have a bias on the $f_{s}$ value, if it has a higher priority than mobiles when reacting to a user request.

When broadcasting its message, each device waits for *d* time to reduce the collision possibility on the shared channels. By default, the delay time is chosen uniformly at random as

(2) $$f_{d}\left(\cdot \right)=U\left[0,d_{w}\right]$$

where $d_{w}$ is the maximum wait delay and U[·] denotes uniform distribution. We show how to determine adaptively $f_{d}$ values according to the given score *k* in Section 3.3.

### 3.2. Framework Parameter Derivation

Here, we derive two framework parameter values, the channel size $c_{size}$ and the maximum wait delay $d_{w}$. In doing so, we first, given the time constraint TC associated with the round, derive the feasible ($c_{size}$, $d_{w}$) pairs. Notice that TC is the time constraint from the event detection to the end of the event processing, and thus TC can be the value obtained by subtracting the event detection delay ($D_{e}$) value from the total time latency requirement (1.2 s) which is specified by the application; each device determines whether to respond within TC = 1.1 s upon the hotword event detection. For broadcasting a message successfully within TC, it should hold that $TC\approx D_{p}+d_{w}+d_{t}$ where $D_{p}$ is the time to perform carrierSensing() once. $d_{w}$ is stipulated by the message transmission time $d_{t}$. The number of usable frequencies per channel can be calculated as $\frac{F_{all}-F_{g}\left(c_{size}-1\right)}{c_{size}}$.

(3) $$\#blocks=1+\lfloor log_{\left\lfloor \frac{F_{all}-F_{g}\left(c_{size}-1\right)}{c_{size}}-1\right\rfloor }S\rfloor$$

(4) $$d_{t}=D_{p}\times \left(\#blocks+1\right)$$

(5) $$d_{w}\approx TC-D_{p}\left(3+\lfloor log_{\left\lfloor \frac{F_{all}-F_{g}\left(c_{size}-1\right)}{c_{size}}-1\right\rfloor }S\rfloor \right)$$

Equation (3) computes the number of blocks for a message, and derives Equation (4) for $d_{t}$ where the transmission time for a block $D_{p}$ is 0.1 s, as explained in Section 2.2. The number of different scores *S* is specified by the application requirements, e.g., 10 in our example. Subsequently, Equation (5) is derived by the time constraint TC above, and then $d_{w}$ can be represented as a function of $c_{size}$ since the other variables in the equation are all constant.

**Figure 8:**
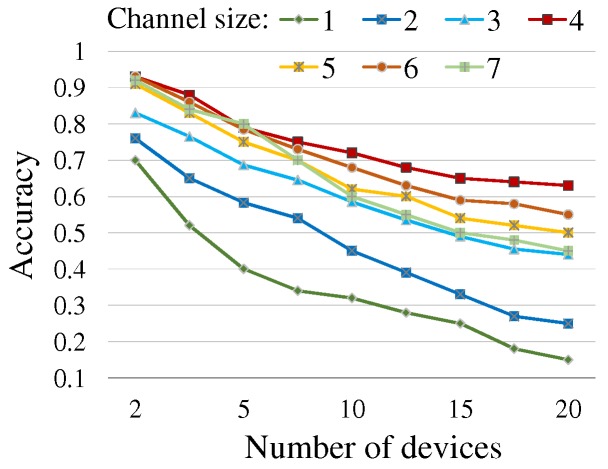
Accuracy by channel size: the graph shows the accuracy (on *y*-axis) that is the rate of successfully resolving a single responder in a group within the time constraint, with respect to the number of devices in a group (on *x*-axis). The simulation experiment was performed 100 times for each pair of channel size and group size.

Table 2 lists the feasible ($c_{size}$, $d_{w}$) pairs derived by Equation (5) with the constraint such that the number of usable frequencies per channel should be no fewer than three; FSK needs at least two frequencies and the *tailtag* message format requires an additional frequency for the 1-bit start tag. Now, to establish the optimal setting, we find the configuration that minimizes the chance of message collisions and thus enables improving the success rate of timely reaching the consensus about the responder. We name the success rate simply *accuracy*. More than a single message being simultaneously sent on the same channel might incur a collision, and thus large $c_{size}$ and $d_{w}$ values can reduce the collision possibility and improve the accuracy. However, the constraint of Equation (5) restricts their valid range. Figure 8 shows the accuracy yielded by simulation experiment with various $c_{size}$ values listed in Table 2 (and the derived valid $d_{w}$ values accordingly) with respect to the size of a device group 2≤N≤20. We then establish the channel size $c_{size}$ = 4 and accordingly $d_{w}$ = 0.7 s as in Table 2, which together yield consistently the better accuracy regardless of the group size. The simulation experiment was performed using the Java-based simulator that we implemented for analyzing the multiple device communication over sound with the *tailtag* framework.

### 3.3. Adaptive Parameters

For improving the framework quality, we propose the adaptive messaging schemes by which the wait delay *d* for broadcasting a message is adaptively determined according to the importance degree of the message specified by the score.

We first consider the score-based delay function of k∈{1,2,…,S} in Equation (6), which yields relatively longer delays for low-scored messages. Intuitively, this intends for sending high-score messages first as well as reducing their collisions, hence being likely to suppress the transmission of unnecessary low-scored messages.

(6) $$\begin{array}{c} f_{d,score}\left(k\right)=\min\left(D_{p}\cdot \left(S-k\right),\;d_{w}\right) \end{array}$$

Given an event, its event detection time might be sightly different among the group devices as the devices individually process the event. Here, we extend the score-based delay function to reflect such asynchronous event detection. Specifically, we employ the adaptive delay by exploiting the received messages of which the transmission time can be presumed according to the score, and the temporal difference estimate δ between the event detection delay of a device and the framework parameter $D_{e}$ value, which the average event detection delay of a group normally follows:

(7) $$\begin{array}{c} f_{d,adapt}\left(k\right)=\min\left(\max\left(f_{d,score}\left(k\right)+\delta ,\;0\right),\;d_{w}\right) \end{array}$$

**Figure 9:**
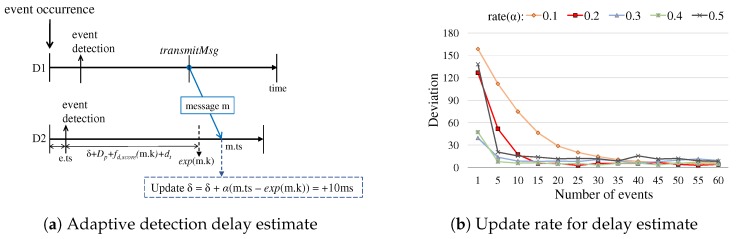
Adaptive mechanism of event detection delay: (**a**) an example of how to update the temporal difference δ between the event detection delay of a device and the framework parameter $D_{e}$, the expected detection delay, using the message *m* and its score *k* received; and (**b**) the impact of α values, different update rates, along with the event occurrences over time. The simulation experiment was performed over 60 subsequent event occurrences for each update rate.

For each device, the δ estimate is continuously updated along with event occurrences and messages as

(8) $$\begin{array}{c} \delta =\delta +\alpha \cdot (m.ts-\left(e.ts+\delta +D_{p}+f_{d,score}\left(m.k\right)+d_{t}\right)) \end{array}$$

where α is the update rate. Figure 9a illustrates an example of adaptive delay updates. Upon an event occurrence, each device individually initiates processing of the event. When a message with the score *k* is received, the device adapts its δ and accordingly its delay function by using the temporal difference between the expected time and the actual time of the message received (denoted by exp(m.k) and m.ts in the figure). This adaptation is effective since we observe through the experiments that the propagation delay of short-range sound-based data communication in the room area context is normally more stable than the event detection delay. Having the simulation results in Figure 9b that shows the derivations of δ values of the group members over the stream of event occurrences, we set the update rate α = 0.2 by default for the stable convergence.

Recall that the framework parameter TC is derived as part of the application requirements. Having high variances on event detection delays among individual devices, however, it is beneficial for each device to continuously calculate the delay-aware time constraint $TC_{\delta }=\min\left(\max\left(TC_{\delta }+\delta ,\;TC-d_{w}\right),\;TC+D_{e}\right)$ and use $TC_{\delta }$ in Algorithm 1 instead of TC.

As explained, a higher-scored message can suppress the transmission of any lower-scored messages and thus it is important that high-scored messages are quickly scheduled. Moreover, it is also beneficial to incorporate the possible ranking of scored messages into the scheduling policy. In the following, we discuss the prediction model in which the wait delay of a *k*-scored message is determined based on the expectation such that *k* is the highest in a group. This intends for not only increasing the chance that the presumably top-ranking message is sent first with a shorter delay even when its score is not in the high range but also mitigating possible collisions even when several high-scored messages exist. Having the score $k=f_{s}\left(e\right)$ for event *e* in Equation (1), we calculate the predictive ranking-based score by

(9) $$\begin{array}{c} f_{s,rank}^{t}\left(k\right)=\left\{\begin{array}{c} k+\left\lfloor \left(S-k\right)\cdot \frac{P_{po}^{t}\left(k\right)-P_{pr}\left(k\right)}{1-P_{pr}\left(k\right)}\right\rfloor ,\;\;\mathrm{if}\;P_{po}^{t}\left(k\right)\geq P_{pr}\left(k\right)\\ \max\left(\left\lfloor k\cdot \frac{P_{po}^{t}\left(k\right)}{P_{pr}\left(k\right)}\right\rfloor ,\;1\right),\;\mathrm{otherwise} \end{array}\right. \end{array}$$

where $P_{po}^{t}\left(k\right)$ is the posterior that the score *k* is the highest at the time duration *t* and $P_{pr}\left(k\right)$ is its prior distribution. The posterior distribution can be used for implementing the candidate() function in Algorithm 1. Note we have the score size *S* as a known parameter and set $P_{pr}\left(k\right)=\frac{k^{N}-{\left(k-1\right)}^{N}}{S^{N}}$ for the group size *N*. Each device continuously updates $P_{po}^{t}\left(k\right)$ exploiting its observations about its scores and the highest scores in its groups at the time duration *t* as:

(10) $$P_{po}^{t}\left(k\right)=\left\{\begin{array}{c} \left(1-\beta \right)P_{po}^{t-1}\left(k\right)+\beta \cdot \frac{\#o^{t}\left(h,k\right)}{\#o^{t}\left(k\right)},\;\;\mathrm{if}\;\#o^{t}\left(k\right)>0\\ P_{po}^{t-1}\left(k\right),\;\;\mathrm{otherwise} \end{array}\right.$$

where $\#o^{t}\left(h,k\right)$ and $\#o^{t}\left(k\right)$ denote the number of observations that the score *k* is given and is the highest, and the number of observations that the score *k* is given, respectively, at the time duration *t*, and β is the update rate. We set the update rate according to the number of observations during the time duration (600 s), i.e.,  $\beta =\min(\max\left(\frac{\sum_{i=1}^{S}\#o^{t}\left(i\right)\cdot TC}{duration(t)},\;0.1\right),\;1.0)$.

## 4. Evaluations

Unless stated otherwise, the configurations of simulation experiments in this section set the default values in Table 1 including the time constraint TC = 1.1 s, the max wait delay $d_{w}$ = 0.7 s, and the channel size $c_{size}$ = 4.

### 4.1. Algorithm Comparison

We discuss the performance of our framework with the different algorithmic strategies introduced in Section 3.3 against various conditions in terms of the event dynamics, the number of devices, and the density in a group. Table 3 lists the conditions. The performance is represented by the accuracy about a single responder with the highest score being resolved successfully within the time constraint for an event. In the experiments, we denote the baseline algorithms with the random delay, base_R (Equation (2)) and with the score-based delay, base_S (Equation (6)). Furthermore, we denote the adaptive algorithms with the adaptive delay, ad_D (Equation (7)) and with the predictive ranking in addition to the adaptive delay, ad_RD (Equation (9)).

**Figure 10:**
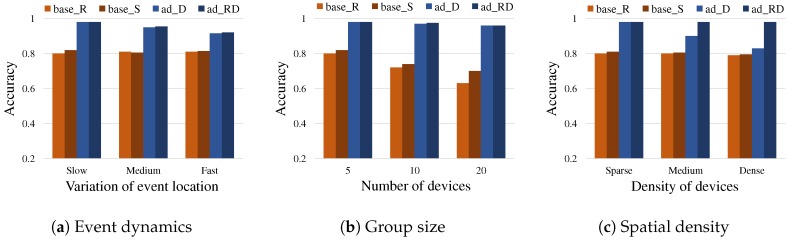
Accuracy by algorithms: having the test conditions in Table 3, the graphs show the accuracy by the algorithms implemented differently using Equations (2) and (6)–(10). The simulation was performed 1000 times for each pair of configuration and algorithm.

Figure 10a shows the simulation result when the various dynamics regarding the event locations were considered, given that the number of devices in a group was set to 5, and Figure 10b shows the simulation result when various group sizes (the number of devices) were considered, given that the event dynamics was set to slow. The adaptive algorithms ad_D, ad_RD both outperformed the other algorithms and specifically showed about 98% accuracy with five devices and slow dynamics case, which we considered one of the commonly recognized multiple user, multiple device environment settings for the uncoordinated react by hotword scenarios. Figure 10c shows the simulation result when different density settings of a device group were considered, depicting the possible advantage of using the predictive ranking in ad_RD particularly for the cases of having many nearby devices in a dense area. Because ad_RD intends to differentiate the similarly scored devices by using the prediction model, its yielded relatively high distribution on wait delays for the dense case.

### 4.2. Tailtag Adoption

**Figure 11:**
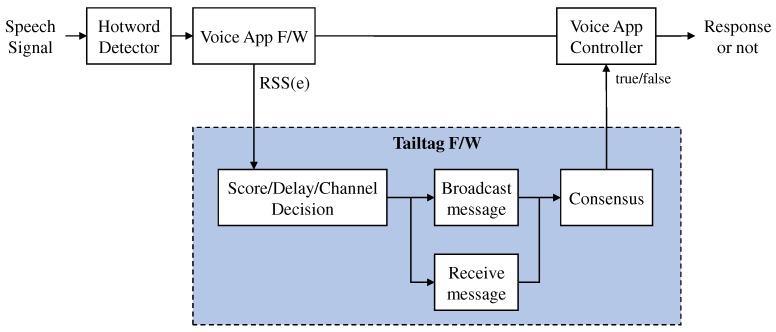
The *tailtag* integration with existing voice app framework.

In most voice assistants, voice commands subsequently issued after a hotword are processed through several tasks including ASR (Automatic Speech Recognition) and NLU (Natural Language Understanding) running on servers, and thus a device needs to keep the dedicated session to the servers for dealing with streams of voice commands. Here, we discuss the prototype implementation of the *tailtag* framework interfacing with Bixby at the device side. As abstracted in Figure 11, the implementation included the two interfaces for processing a hotword signal as an input event to the *tailtag* framework as well as returning the corresponding output.

**Figure 12:**
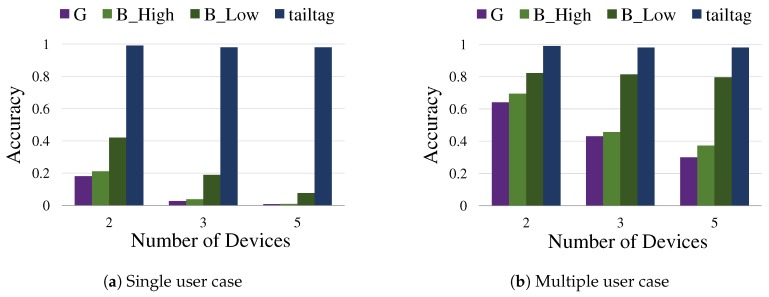
Accuracy by Framework: (**a**) the test result of multiple devices of a single user; and (**b**) the test result of multiple devices of multiple users (where only one device is registered for the user saying the hotword). The *x*-axis denotes the number of devices and the *y*-axis denotes the accuracy. The test was performed 100 times for each combination of five testers, four compared solutions, and the number of devices.

Figure 12 shows the benefit of the coordination by *tailtag* in a multiple device, multiple user environment. We compared Google Assistant (denoted by G) and existing Bixby of different sensitivity settings (denoted by B_High and B_Low) with our *tailtag*-based prototype (denoted by tailtag) in terms of accuracy. The experiment conditions were set as those of Bixby sensitivity tradeoff tests in Figure 2, having Samsung Galaxy S8 devices, five adult testers, and 1 m distance between a tester and devices in the same room setting with insignificant ambient noise. Figure 12a,b depicts the cases of multiple devices of a single user and multiple devices of multiple users, respectively, clearly demonstrating the high accuracy of our approach, no less than 98% when the group size was up to five. Notice that the low accuracy by the existing voice-based services was mostly because they do not normally deliberate much on the implication of multiple devices and multiple users yet, and rarely include the coordination process of devices when processing user requests. Accordingly, we frequently observed redundant responses in the tests. It should be also noted that the tests focused only on the coordinated response to hotwords, and thus the result should be separated from all the other performance factors of voice-based services.

Our prototype managed to meet the feasibility requirements of our target scenario in terms of accuracy and latency, which were initially discussed with the user experience team. In the case of using the existing voice app framework of Bixby, we know empirically that it takes 0.8 s on average to react to hotwords (with no coordination), and, as demonstrated through the experiment with devices, the *tailtag* framework was successfully configured to satisfy the time constraint of 1.1 s (TC) with high accuracy.

## 5. Related Work

Several works exploit sound wave to transmit data among consumer devices. Google Tone [27] allows computer users to broadcast URL data to nearby devices through sound wave. Signal360 [28] enables performing customized services in indoor stores, exploiting beacon signals that are emitted via BLE or inaudible sound wave. Starbucks offers a remote ordering service (namely, siren order) that provides the online ordering and payment method [29]. Inaudible sound was tested for siren order. As explained, it is hard to pinpoint the user location (e.g., whether a user is in the store or not) using BLE, WiFi, or GPS, and thus sound-based data communication becomes a solution for the room area context. While the user scenarios using sound-based data communication above are commonly relevant to one-to-many device communication (e.g., a store server to several consumer devices), our interest is rather on many-to-many (e.g., consumer devices to consumer devices) for enabling the ephemeral group communication.

Chirp SDK [30] is a commercially packaged solution that aims to efficiently handle multi-bytes data payloads and reliably support one-to-one and one-to-many device communication over sound in the domain of consumer device apps, and accordingly its message format contains a relatively large header with error correction symbols. Our internal tests observed that such multi-byte messaging degrades the performance for the ephemeral group scenario that requires small data transmission in many-to-many device communication with the time constraint.

A novel form of real-time sound communication method called Dolphin is proposed in [31]. Experiments on ambient noise measurement show that noise can be ignored at frequencies higher than 8 kHz. Thus, both audible and inaudible bands in 8–20 kHz are used. Dolphin achieves 500 bps at a decoding rate within 1 m distance, but does not consider multiple device environments.

In mobile communication, CDMA enables multiple devices to communicate simultaneously over the entire frequency band. Such multiple access technologies have been seldom researched for sound wave communication. Most relevant efforts have been made for Underwater Wireless Sensor Networks (UWSNs) [32,33], concentrating on the design of contention-based MAC protocols. It is rarely feasible to incorporate the existing contention-based protocols into our framework due to the ephemeral group structure with no controller. Using network coordinators [34], channel reservation [35], or handshaking [36,37,38] over sound wave is less desirable for low latency communication in an ephemeral group.

In a voice-assisted app, keyword spotting system (KWS) runs to quickly detect a trigger phrase (hotword) activating the app. As demonstrated by the experiments previously, this often creates redundant or seemingly arbitrary responses when multiple devices are involved. In [39], the authors proposed a novel KWS that explores the contextual automatic speech recognition, showing improvement such that the false alarm is reduced by 89%. However, they focused on single device cases and rarely considered the environment conditions where several devices are closely located.

When CSMA/CA is applied as a multiple access scheme in inter-vehicular wireless communication, the probability of message collision increases as the vehicle density becomes larger. The work in [40] proposes the adaptive scheme, Distance-Dependent Adaptive Backoff (DDAB), that utilizes the different ranges of random backoff depending on the location of vehicles especially for facilitating the distribution of emergency warning messages efficiently. This work is relevant to the score-based delay of *tailtag* in that both explore the similar concept of priority-based messaging. However, the *tailtag* framework uses the sound-based data communication for consumer devices and explores not only message scheduling but also message suppression based on scores.

Leader election has been studied in the domain of distributed systems [11,12,14,15]. Our approach using broadcast messages to make a brief consensus on the group responder upon an external event can be seen as relevant to bully algorithms [13,16]. Several research works about cluster head selection [41,42,43] in sensor networks also have similar relevance. While sharing the common structure of leader election with those previous works, however, the framework in this paper differently concentrates on two challenging conditions, namely the unstructured group communication over sound and the time constraint on the election process, which render the work here unique as well as applicable to the real world scenarios of voiced-enabled devices.

## 6. Conclusions

The presented framework *tailtag* addresses the issue of possible uncoordinated, redundant responses of smart consumer devices that interact with a user. To do so, we introduce the notion of an ephemeral group, in which a set of devices individually detecting an external event make a group implicitly and communicate shortly for deciding the event responder, and utilize inaudible sound for unstructured group communication with room area context. We view such ephemeral group coordination as the time-constrained leader election process over sound, and employ the importance-aware adaptive messaging in the group to satisfy the given time bound requirement. For dealing with hotwords in voice assistants of consumer devices, we exploit the isomorphic room area capability of detecting hotword events and communicating over sound in a group.

The simulations and the prototype tests of real-world consumer device scenarios demonstrate that, without any prebuilt controller or backend infrastructure for coordination, a set of devices in a close range can communicate short messages and reach a certain consensus efficiently in a timely manner. Thus far, we have shown the feasibility of the framework, focusing on the specific target scenarios of handling a hotword event among multiple devices. Our future work includes adopting the framework more than dealing with a single event and group type, and thus generalizing the framework structure to support variable time constraints and different external event types.

## Figures and Tables

**Table 1:** Framework parameters.

Symbol	Definitions (Default Values)	Decided by
$F_{all}$	Number of available frequencies for the entire bandwidth (100)	Framework design
$F_{g}$	Number of available frequencies for a guard interval (12)	Framework design
$D_{p}$	Transmission time for a block (0.1 s)	Framework design
TC	Time constraint (1.1 s)	App
*S*	Score size (10)	App
$D_{e}$	Event detection delay (0.1 s)	App
$d_{t}$	Transmission time for a message (0.3 s)	Derivation
$d_{w}$	Maximum wait delay (0.7 s)	Derivation
$c_{size}$	Channel size (4)	Derivation

**Table 2:** Feasible configurations.

$c_{\mathrm{size}}$	#blocks	$d_{t}$	$d_{w}$
1–4	2	0.3 s	0.7 s
5–6	3	0.4 s	0.6 s
7	4	0.5 s	0.5 s

**Table 3:** Experiment configuration.

Condition	Definition	Settings
Event dynamics	The average number of successive event occurrences in the same location	slow = 10, medium = 5, fast = 1
Group size	The number of member devices in a group	N = 5, 10, 20
Group spatial density	Average distance between devices (m)	sparse = 2, medium = 1, dense = 0.5
